# Predicting lesion reversal in acute cerebral ischaemia via apparent diffusion coefficient threshold on diffusion-weighted MRI

**DOI:** 10.1007/s00330-025-12014-0

**Published:** 2025-09-29

**Authors:** Thor Håkon Skattør, Atle Bjørnerud, Terje Nome, Kine Mari Bakke, Brian Anthony Enriquez, Ingrid Digernes, Cecilie Mørck Offersen, Mona Kristiansen Beyer, Geir Ringstad, Anne Hege Aamodt

**Affiliations:** 1 Division of Radiology and Nuclear Medicine, Oslo University Hospital, Oslo, Norway; 2 Department of Neurology, Oslo University Hospital, Oslo, Norway; 3 Institute of Clinical Medicine, University of Oslo, Oslo, Norway; 4 Department of Physics and Computational Radiology, Oslo University Hospital, Oslo, Norway; 5 Centre for Lifespan Changes in Brain and Cognition, University of Oslo, Oslo, Norway; 6 Department of Radiology, Copenhagen University Hospital, Rigshospitalet, Copenhagen, Denmark; 7 Department of Clinical Medicine, University of Copenhagen, Copenhagen, Denmark; 8 KG Jebsen Centre for Brain Fluid Research, University of Oslo, Oslo, Norway; 9 Department of Neuroscience and Movement Science, The Norwegian University of Science and Technology, Trondheim, Norway

**Keywords:** Brain, Diffusion magnetic resonance imaging, Endovascular procedures, Ischaemic stroke, Reperfusion

## Abstract

**Objectives:**

Diffusion-weighted imaging (DWI) quickly detects early ischaemic changes, but does not necessarily signify irreversible tissue damage, as DWI lesion reversal (DWI-R) can occur. Apparent diffusion coefficient (ADC) thresholds have been proposed to distinguish salvageable from irreversibly damaged tissue. This study aimed to evaluate the predictive value of a single ADC threshold for DWI-R following rapid and successful recanalization with voxel-level methodology.

**Materials and methods:**

In this cohort study, we retrospectively analysed consecutive patients examined with DWI before and the day after endovascular therapy, with successful recanalization within 120 min of baseline MRI. DWI-R was assessed voxel-wise for ADC values between 200 mm^2^/s and 760 × 10^−6^ mm^2^/s. Predictive accuracy of ADC thresholds was evaluated using receiver operating characteristic (ROC) analyses.

**Results:**

Seventy-one patients with a mean baseline DWI lesion volume of 18.13 mL (IQR: 6.15, 26.25) were included. Median time from MRI to recanalization was 84.0 min (IQR: 72.0, 95.5). On average, 37.5% of voxels demonstrated reversal. The area under the curve for predicting reversal based on ADC was 0.708, and the optimal threshold was 555 × 10^–6^ mm^2^/s (sensitivity 73.8%, specificity 58.6%). The voxel-wise probability of reversal declined with lower ADC, but even low ADC values exhibited some degree of reversal.

**Conclusion:**

This study reinforces existing concerns about using fixed ADC thresholds to define irreversible injury. A single ADC cut-off showed only modest sensitivity and poor specificity for predicting DWI-R. No definitive lower ADC boundary was identified across clinically relevant ranges below which the likelihood of DWI-R became negligible.

**Key Points:**

***Question*** *Identifying penumbra from permanent ischaemic damage remains challenging, and the role of ADC evaluation before thrombectomy in predicting tissue viability is debated*.

***Findings*** *An ADC threshold of 555 × 10−6 mm²/s yielded moderate sensitivity (73.8%) and low specificity (58.6%) for predicting DWI-R (AUC 0.708)*.

***Clinical relevance*** *A uniform ADC threshold has limited utility in identifying salvageable brain tissue in thrombectomy triage. Our findings emphasise the need for caution when excluding acute ischaemic stroke patients from recanalization therapy based on restrictive diffusion*.

**Graphical Abstract:**

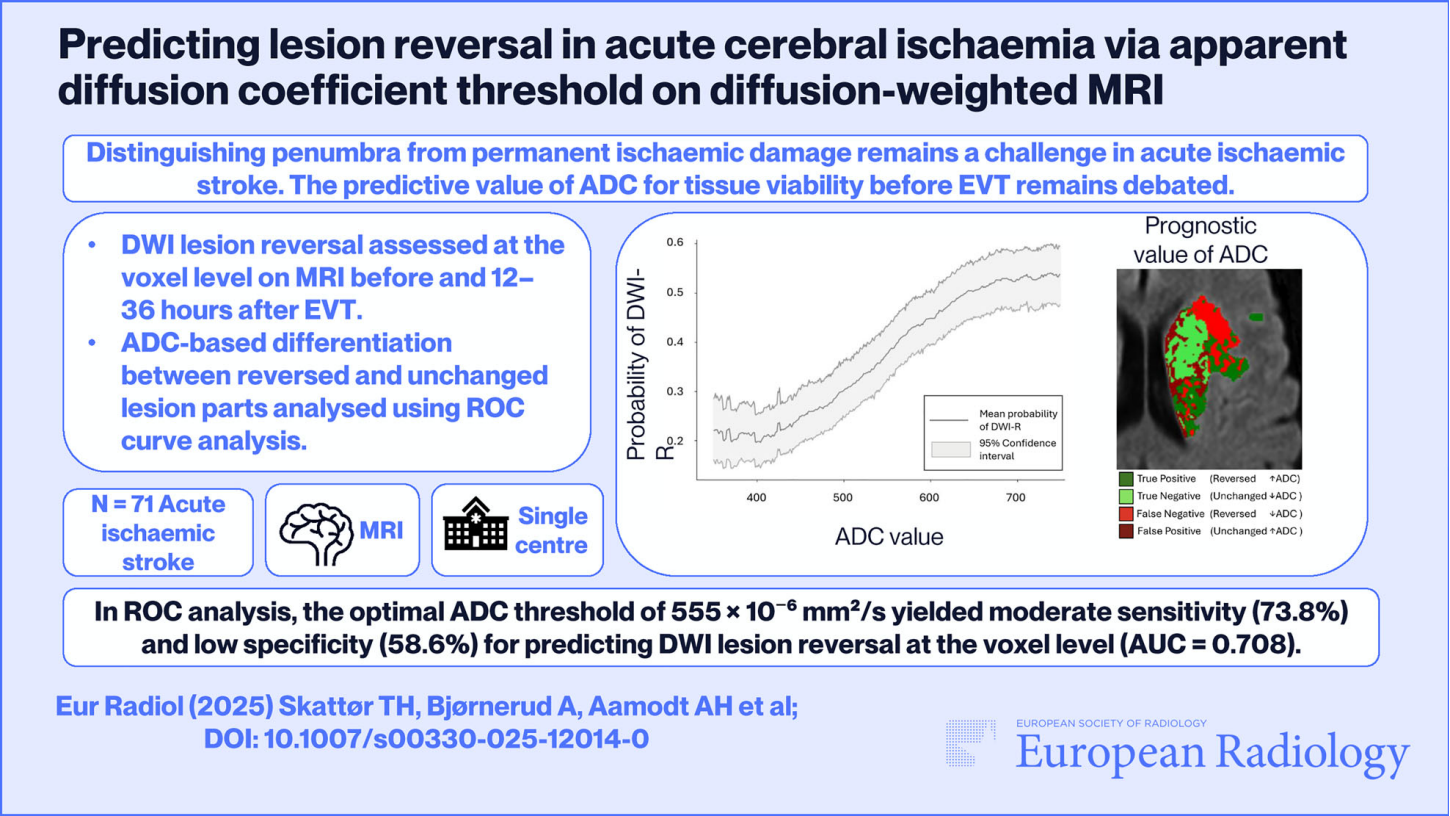

## Introduction

Rapid and accurate assessment of ischaemic lesion extent is crucial in acute stroke management, informing decisions about endovascular therapy (EVT) and other reperfusion therapies. Diffusion-weighted imaging (DWI) obtained with MRI, has become a key diagnostic tool for early detection of ischaemic changes, as it is sensitive to the restriction of water diffusion associated with cellular injury [1–3]. However, increasing evidence shows that DWI hyperintensities are not always a definitive marker of irreversible infarction. Reversal of DWI lesions (DWI-R) has been documented, especially in patients where reperfusion is achieved [4]. The apparent diffusion coefficient (ADC) is a critical indicator of tissue viability, with lower ADC values reflecting more severe ischaemic injury [5].

Numerous studies have used ADC for distinguishing irreversibly damaged “core” tissue from potentially salvageable “penumbral” regions [5–10]. A frequently cited threshold is 620 × 10^–6^ mm^2^/s [9]. However, normal ADC measurements vary substantially across the brain: grey matter exhibits higher ADC values than white matter, and ADC may differ in superficial cortical layers versus deeper structures [11]. These physiological differences, compounded by potential hardware and sequence variations across imaging platforms [12] and post-processing software [13], complicate the establishment of a universal threshold. Importantly, over-reliance on a single ADC threshold may carry clinical risks. Patients with salvageable brain tissue could be misclassified as having irreversible infarction, potentially leading to inappropriate exclusion from reperfusion therapies. This concern is relevant in the broader context of stroke imaging. While CT-based perfusion imaging is widely available, DWI remains the reference standard for defining infarct core and is often used to validate perfusion imaging techniques [14–16]. Thus, a nuanced understanding of ADC thresholds is essential for accurate stroke imaging interpretation and for guiding informed clinical decision-making.

The objective of this study was to examine the predictive value of a straightforward, voxel-based ADC threshold for DWI-R in patients achieving rapid and successful recanalization by EVT. By comparing baseline DWI to follow-up imaging after EVT, we assessed whether a single “optimal” threshold could effectively distinguish salvageable from unsalvageable tissue. Additionally, we aimed to determine whether a lower ADC boundary exists below which the likelihood of lesion reversal becomes negligible.

## Materials and methods

Ethical clearance was granted by the regional committee (REK 2015/1844). Written informed consent was obtained from all participants or, when necessary, from their legally authorised representative. The study was conducted in accordance with the principles of the Declaration of Helsinki. The Oslo Acute Reperfusion Stroke Study (OSCAR), NCT06220981, is a prospective observational cohort study on acute ischaemic stroke patients who are receiving EVT at Oslo University Hospital, Oslo, Norway. We performed a retrospective analysis of OSCAR patients who were included from January 2017 to March 2022. Inclusion criteria for the current study encompassed MRI scans obtained before EVT and repeat imaging 12–36 h after EVT. Exclusion criteria encompassed a lack of standardised DWI sequence of sufficient quality, no initial DWI lesion, insufficient quality of recanalization measured as modified treatment in cerebral infarction score (mTICI) < 2c, long time to recanalization >2 h after MRI, and re-occlusion of any treated vessels [17, 18]. The 2-h threshold was selected to balance the risk of additional cellular injury within the ischaemic lesion prior to reperfusion against the need to maintain an adequate sample size for analysis. Data collection was limited to patients treated up to March 2022 to mitigate selection bias, as MRI use in EVT patients became less routine and more restricted to complex cases.

### Imaging and image post-processing

DWI was acquired on two identical 1.5-Tesla Siemens MAGNETOM^®^ Aera scanners using a single-shot, echo-planar imaging (EPI) sequence with three-directional diffusion encoding and trace-weighted reconstruction. Imaging parameters included a voxel size of 0.6 × 0.6 × 6.5 mm, an echo time of 90 (±2 ms), a repetition time of 6400 (±700 ms), and a matrix size of 384 × 384. ADC was calculated from images acquired at *b*-values of 0 s/mm², 500 s/mm², and 1000 s/mm², and lesion segmentation was performed on calculated b1500 maps to enhance lesion visibility and segmentation accuracy [19]. DWI lesions were segmented using the NordicICE software [20] and ADC maps as reference. In cases of uncertainty, an ADC difference of ≥ 100 × 10^–6^ mm^2^/s from the contralateral side was used as the criterion for inclusion of the DWI lesion. Post-EVT masks were co-registered to pre-EVT DWI images with ANTsPy [21]. Voxels included on pre-EVT masks, but not on post-EVT masks, were categorised as DWI-R, while voxels included on both masks were categorised as Unchanged. On pre-EVT images, voxels with higher ADC value than 760 × 10^–^^6^ mm^2^/s were excluded, and voxels below 200 were considered artifacts or inaccuracies in segments and excluded. Voxels of all ADC values were included in the post-EVT lesion masks to avoid overestimation of DWI-R. Regions with haemorrhagic transformation were included on the post-EVT scans (but not pre-EVT scans) and were therefore conservatively classified as persistent infarction. Since ADC values were measured exclusively on the pre-EVT MRI, haemorrhage-related signal changes did not influence the ADC analysis. To mitigate the risk of overestimating DWI-R in small lesions, where inaccuracies in segmentation and co-registration may result in a disproportionately high portion of the lesion being misclassified, we included only patients with pre-EVT DWI lesion volumes greater than 1 mL.

### Reliability of the segmentation process

Seventy consecutive DWI studies were independently segmented by two experienced interventional neuroradiologists (T.H.S., T.N.), each with more than 10 years of experience in EVT, clinical neuroimaging, and stroke MRI interpretation. Both were blinded to clinical and procedural information. The inter-rater agreement, quantified using the Dice coefficient, was strong at 0.852 (95% CI: 0.834–0.880). In cases where the Dice coefficient fell below 0.7, discrepancies were addressed through consensus. Following this process, a single rater (T.H.S.) segmented the remaining dataset, and these segmentations were used for the final analyses.

### Variables under consideration

ADC values and volumes of DWI-R and unchanged lesion areas were calculated based on pre- and post-EVT DWI lesion masks applied to pre-EVT ADC maps. The mTICI scores were evaluated by an interventional neuroradiologist blinded to the initial rating from the patient journal. Occluded vessel, DWI-ASPECTS score [22], and Heidelberg bleeding classification [23] status were evaluated on radiological images. Time for ictus was obtained from a medical journal. Time for MRIs and recanalization was collected from image metadata. Pre-stroke modified Rankin Scale (mRS), NIHSS [24], and status of thrombolysis were collected from medical records. Three-month mRS was obtained from clinical visits or phone interviews [25].

**Statistical analyses** were performed using SciPy version 1.11.4 and STATA version 18.0. Volumes, ADC values, and times were treated as continuous variables, while DWI-R was treated as a binary variable at the voxel level. The predictive value of ADC thresholds ranging from 200 to 760 × 10^−6^ mm²/s was assessed using receiver operating characteristic (ROC) analysis. The optimal threshold was determined based on Youden’s J statistic. A subgroup analysis was performed on patients with anterior circulation occlusions and mTICI 3 recanalization within 90 min from MRI acquisition. To evaluate the probability of DWI-R at different ADC levels, voxel-wise probability calculations were conducted both at the individual patient level and for the dataset. In this probability analysis, the proportion of reversed voxels within a given ADC range (ADC ± 10 × 10^−6^ mm²/s) was calculated by dividing the number of reversed voxels in that range by the total number of voxels within the same range. For comparisons of numerical variables, a student’s *t*-test was applied for normally distributed data, whereas the Mann–Whitney *U*-test was used for non-normally distributed data.

## Results

**Fig. 1:**
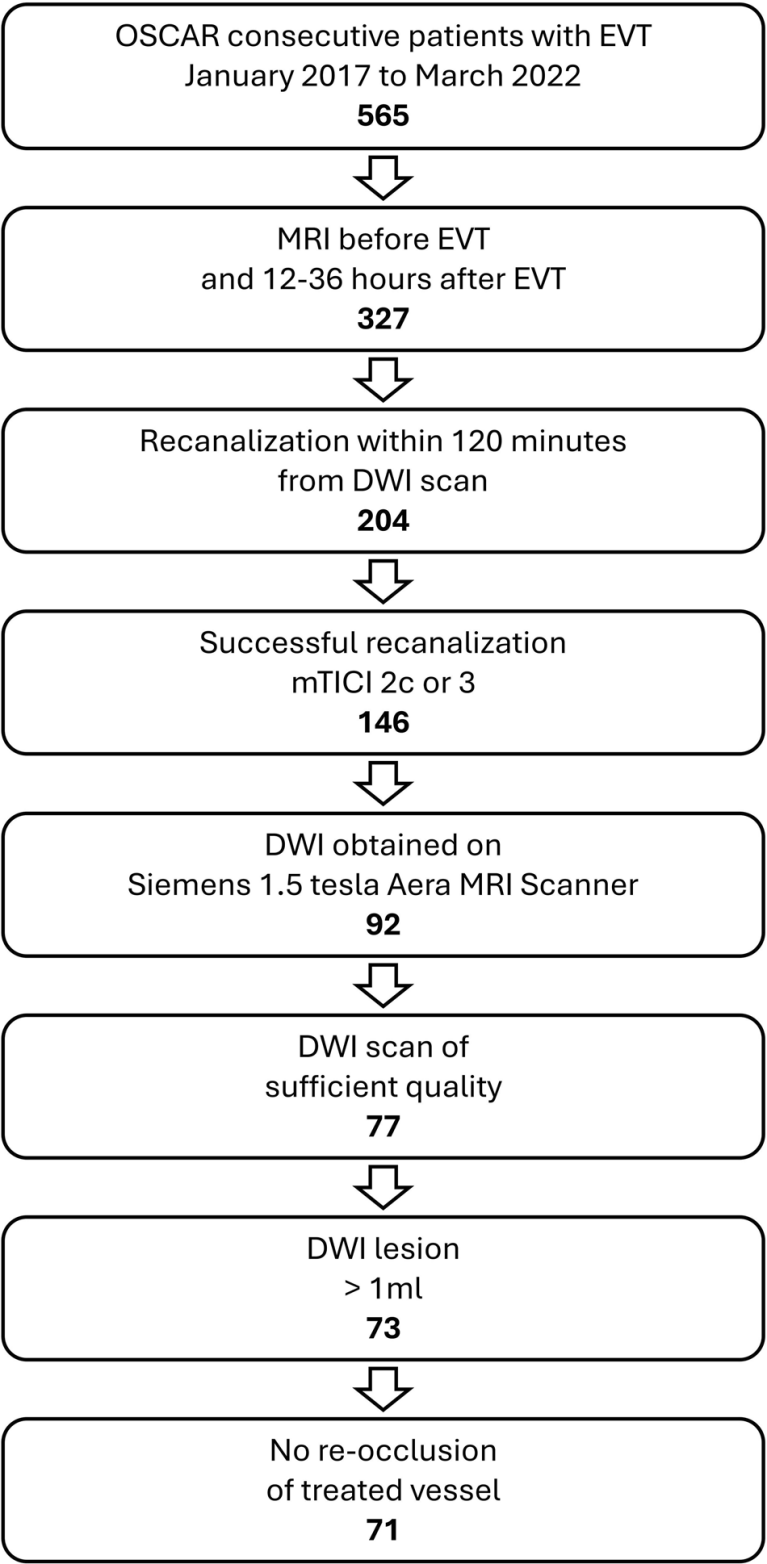
Study inclusion flow diagram

A total of 565 patients were included in the OSCAR study during the inclusion period. Among them, 204 underwent MRI both before and after EVT within the specified time frame. Successful reperfusion, defined as an mTICI grade of 2c or 3, was achieved in 146 of these patients. In 92 of these cases, DWI scans were obtained with the same sequence type both before and after EVT using one of two equally equipped Siemens Aera 1.5-Tesla scanners. Sufficient image quality for lesion analysis and co-registration was available for 77 patients. A pre-EVT DWI lesion of at least 1 mL was found in 73 patients. Two patients were excluded due to re-occlusion of the treated vessel, leaving 71 patients for final inclusion. A study inclusion flow diagram is shown in Fig. 1. Demographics, baseline characteristics, and procedural information of the included patients are provided in Table 1. Information on additional MRI sequences acquired pre- and post-EVT is provided in Supplementary Table S1.

**Table 1:** Basic characteristics

Variables	*N* = 71	Missing
Sex, male (%)	31 (43.7)	0
Age, median years (IQR)	72.0 (61.5, 79.5)	0
Occlusion type		0
LVO (%)	48 (67.6)	
MeVO (%)	18 (25.4)	
Posterior circulation (%)	5 (7.0)	
Thrombolysis (%)	49 (69.0)	0
NIHSS, median (IQR)	12.0 (8.0, 18.0)	0
Time Ictus-MRI, median min (IQR)	219.0 (176.0, 286.5)	0
Time MRI—Recanalization, median min (IQR)	84.0 (72.0, 95.5)	0
Time Ictus—Recanalization, median min (IQR)	303.0 (253.0, 363.5)	0
Time Recanalization—MRI-post, median hours (IQR)	21.6 (19.3, 24.5)	0
DWI-ASPECTS pre-EVT, median (IQR)	7.0 (6.0, 8.0)	0
DWI-ASPECTS pre-EVT, median (IQR)	7.0 (6.5, 8.0)	0
mTICI 2c (%)	37 (52.1)	0
mTICI 3 (%)	34 (47.9)	0
EVT technique		0
Stentretriever (%)	59 (83.1)	
Aspiration only (%)	6 (8.5)	
Stentretriever + carotid artery stent (%)	3 (4.2)	
Stentretriever + intra-arterial thrombolysis (%)	1 (1.4)	
Spontaneous recanalization (observed on DSA) (%)	2 (2.8)	
Heidelberg bleeding classification		0
1a/b petechial (%)	22 (31.0)	
1c parenchymal small (%)	11 (15.5)	
2 parenchymal mass effect (%)	4 (5.6)	
3c subarachnoid (%)	3 (4.2)	
Pre-stroke mRS ≤ 2 (%)	68 (95.8)	0
3-months mRS ≤ 2 (%)	58 (82.9)	1

Demographics and basic characteristics of included patients. Values are given in number of patients and percentage in parentheses unless otherwise specified

*LVO* large vessel occlusion, *MeVo* medium vessel occlusion, *NIHSS* NIH stroke scale, *IQR* interquartile range, *DWI-ASPECTS* diffusion-weighted imaging-Alberta stroke program early CT score, *EVT* endovascular therapy, *mTICI* modified treatment in cerebral infarction score, *mRS* modified Rankin scale

**Fig. 2:**
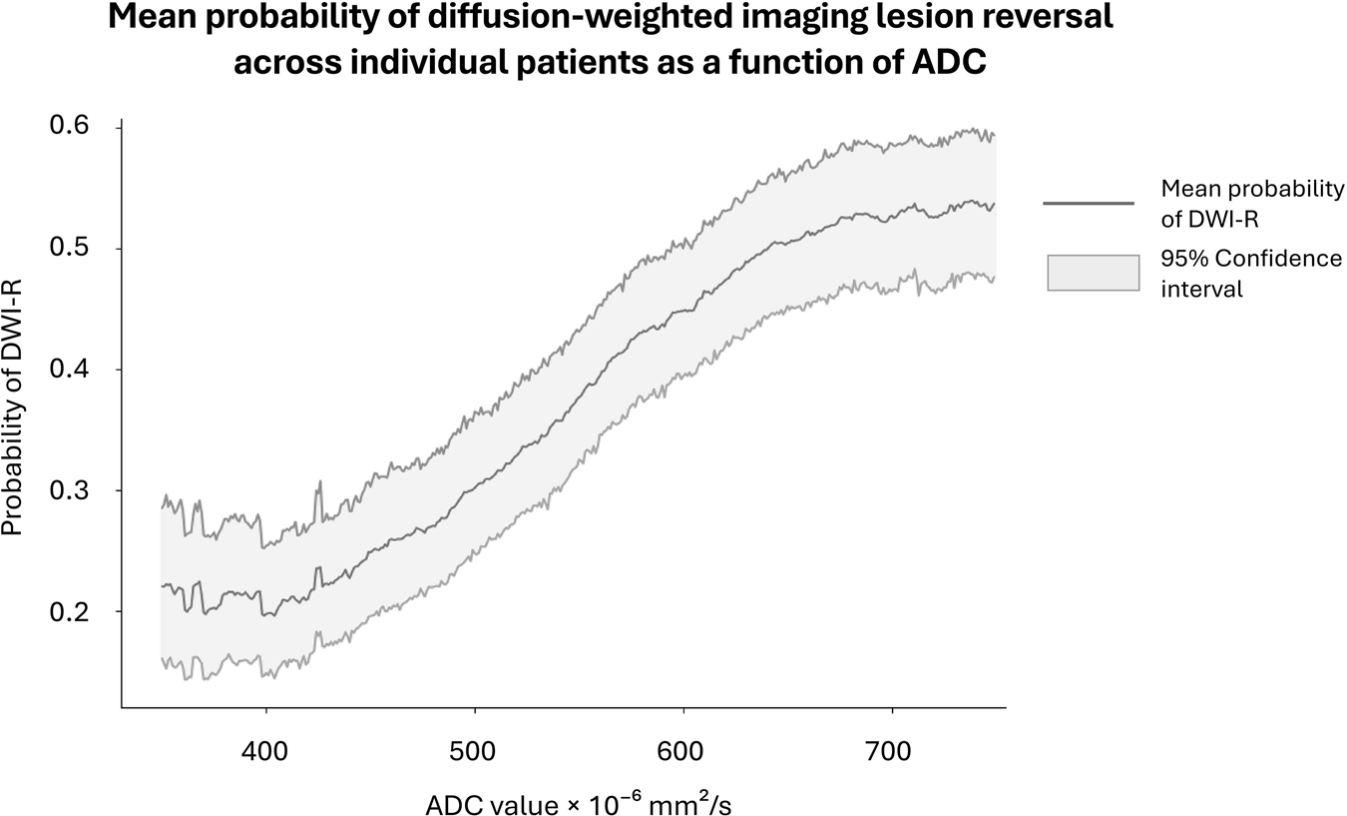
This figure presents the probability for voxel reversal at various ADC levels. The graph represents the mean probability for voxels across individual included patients at a given ADC level ±10 × 10^−6^ mm²/s in the range of 350–750 × 10^−6^ mm²/s

The mean DWI lesion volume was 18.13 mL (IQR: 6.15, 26.25). The mean volume of DWI-R was 6.77 mL (95% CI: 4.85, 9.00), which corresponds to 37.3% of the initial lesion volume. The mean ADC value in reversed lesion segments was 597.6 × 10^−6^ mm²/s (95% CI: 587.2–607.6), compared with 536.7 × 10^−6^ mm²/s (95% CI: 523.9– 550.2) in the unchanged segments, yielding a difference of 60.9 × 10^−6^ mm²/s (*p* < 0.001). The proportion of DWI-R across ADC thresholds ranging from 250 × 10^−6^ mm²/s to 750 × 10^−6^ mm²/s is presented in Table 2, alongside the volumes of DWI-R and the total DWI lesion. The probability of DWI-R for voxels at various ADC levels is shown in Table 2 and Fig. 2. Higher ADC values were generally associated with an increased probability of lesion reversal.

**Table 2:** Lesion reversal across ADC thresholds

ADC threshold ×10^−6^ mm²/s	DWI lesion volume below threshold in mL (% of total DWI lesion)	DWI-R volume below threshold in mL (% of total DWI-R)	Proportion of DWI-R in voxels below threshold in %	Sensitivity in %	Specificity in %	Probability of DWI-R for voxels with this ADC value ±10 × 10^−6^ mm²/s in %
750	17.85 (98.5)	6.61 (97.6)	37.03	2.68	98.94	59.44
700	16.03 (88.4)	5.53 (81.6)	34.47	18.81	92.38	58.89
650	13.68 (75.5)	4.20 (62.0)	30.68	38.43	83.33	53.14
620	12.12 (66.9)	3.39 (50.0)	27.96	50.38	76.63	49.30
600	11.02 (60.8)	2.86 (42.2)	25.96	58.16	71.59	46.54
555	8.50 (46.9)	1.80 (26.5)	21.17	73.78	58.64	36.95
550	8.21 (45.3)	1.70 (25.0)	20.65	75.26	57.07	35.82
520	6.59 (36.3)	1.17 (17.2)	17.72	83.01	47.35	29.03
500	5.54 (30.6)	0.88 (13.0)	15.92	87.17	40.66	25.43
450	3.24 (17.9)	0.40 (5.9)	12.29	94.22	24.72	17.39
400	1.61 (8.9)	0.16 (2.4)	9.93	97.68	12.57	11.30
350	0.68 (3.7)	0.06 (0.9)	8.68	99.15	5.32	9.88
300	0.23 (1.3)	0.02 (0.3)	8.05	99.73	1.82	7.38
250	0.06 (0.3)	0.00 (0.1)	8.14	99.94	0.43	7.36

Voxel-based analysis of DWI lesion reversal (DWI-R) across ADC thresholds (250–750 × 10^−6^ mm²/s). For each threshold, the table shows: the mean volume of DWI lesion voxels with ADC values below the threshold, the mean volume of those voxels that reversed after endovascular treatment, the proportion of all reversed voxels found below the threshold, voxel-level sensitivity and specificity for detecting DWI-R, and the probability of reversal for voxels with that specific ADC value (±10 × 10^−6^ mm²/s)

**Fig. 3:**
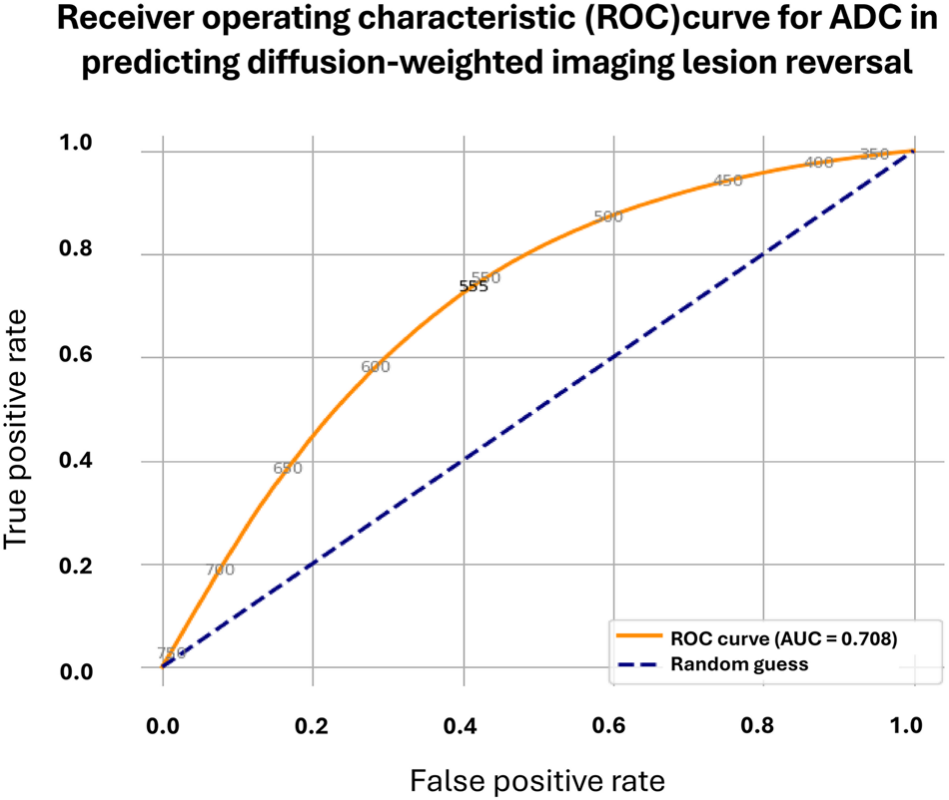
ROC curve for ADC in predicting DWI-R at the voxel level, within the ADC range of 200–760 × 10^−6^ mm²/s

**Fig. 4:**
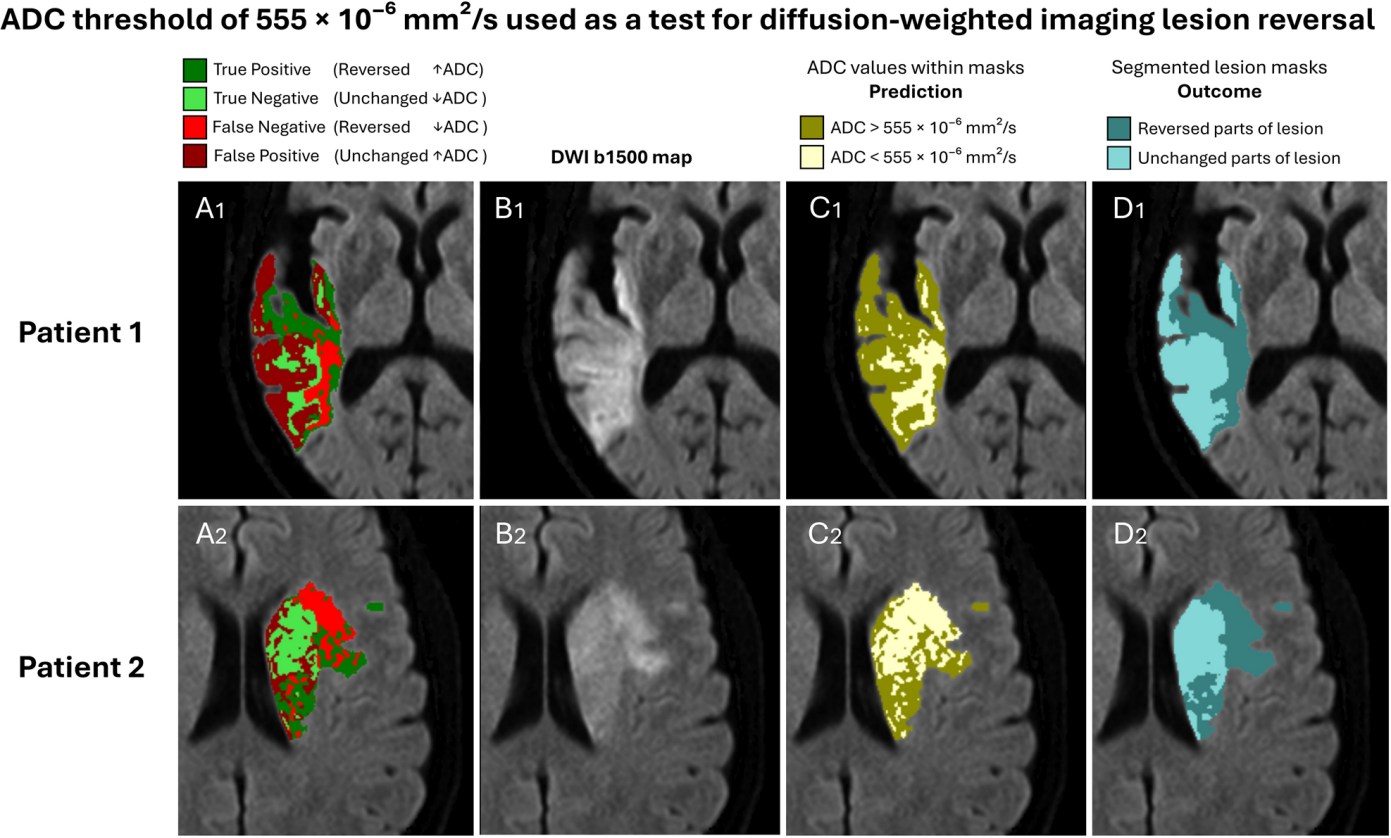
Illustration of voxel-level ADC as a predictive test for DWI-R on b1500 maps in two patients. The optimal threshold in our dataset for distinguishing voxels that would reverse from those that would not was ADC 555 × 10^−6^ mm²/s. **A** shows the test result, **B** shows the plain DWI lesion, **C** shows the applied test (ADC level above vs below the threshold), and **D** shows the true outcome (reversed vs unchanged). Voxels above the threshold are shown in dark colours (**A**, **C**), while voxels below are shown in light colours. Voxels are also colour-coded based on the true outcome (**A**): dark green: true positive (reversed voxels with high ADC values), light green: true negative (unchanged voxels with low ADC values), light red: false negative (reversed voxels with low ADC values), and dark red: False positive (unchanged voxels with high ADC values)

**Fig. 5:**
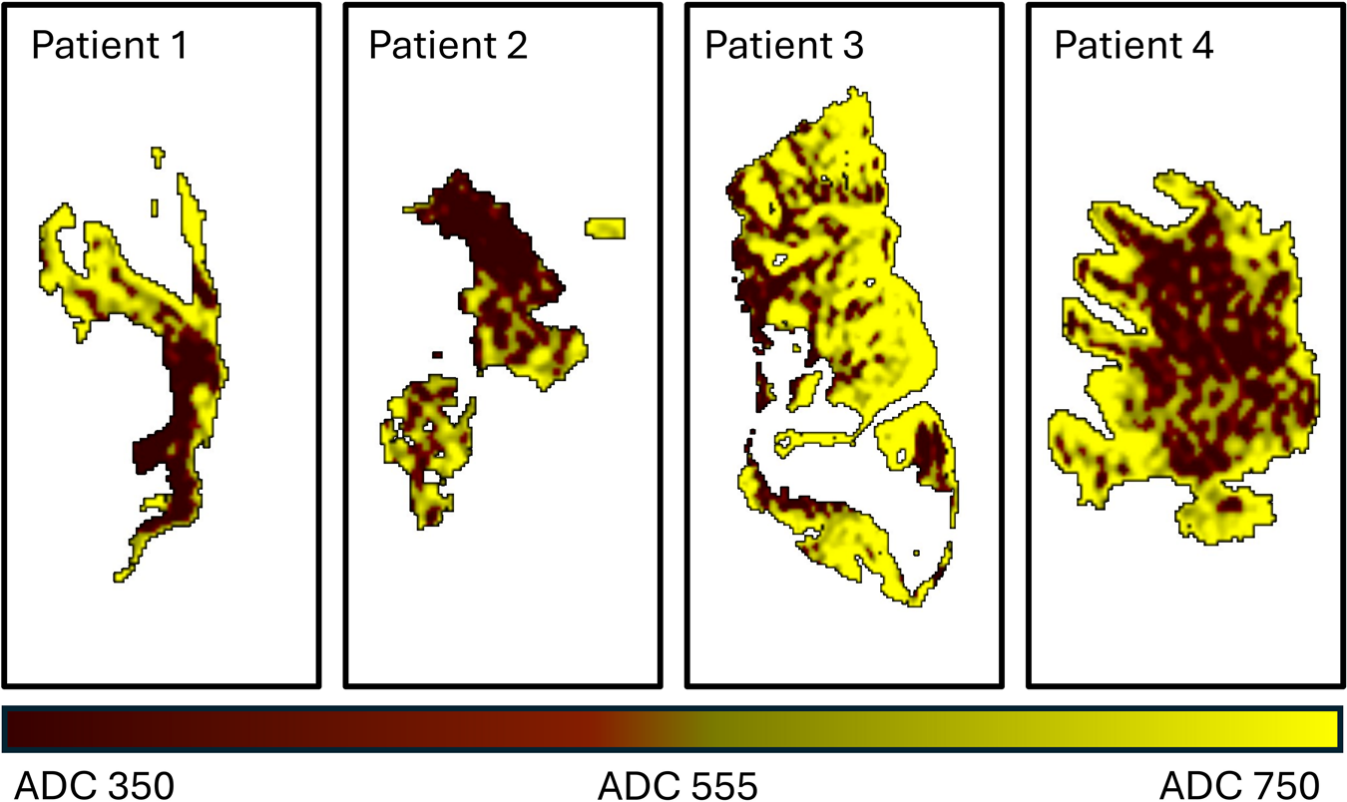
Illustration of ADC levels in the reversed lesion parts of four patients. Lesion areas with ADC below 555 × 10^−6^ mm²/s, identified as the optimal threshold for distinguishing reversed from unchanged voxels in our study, are shown in shades of red, with darker shades indicating lower ADC values. Voxels with ADC above the threshold are coded in yellow. This illustration highlights the broad range of ADC values observed in reversed lesion areas. In Patients 1, 3, and 4, the underlying structure of grey matter (which typically has higher ADC values in the normal brain) and white matter (with lower ADC values) is visible, demonstrating how these anatomical differences translate into the ADC values seen in diffusion-weighted imaging lesions

**Fig. 6:**
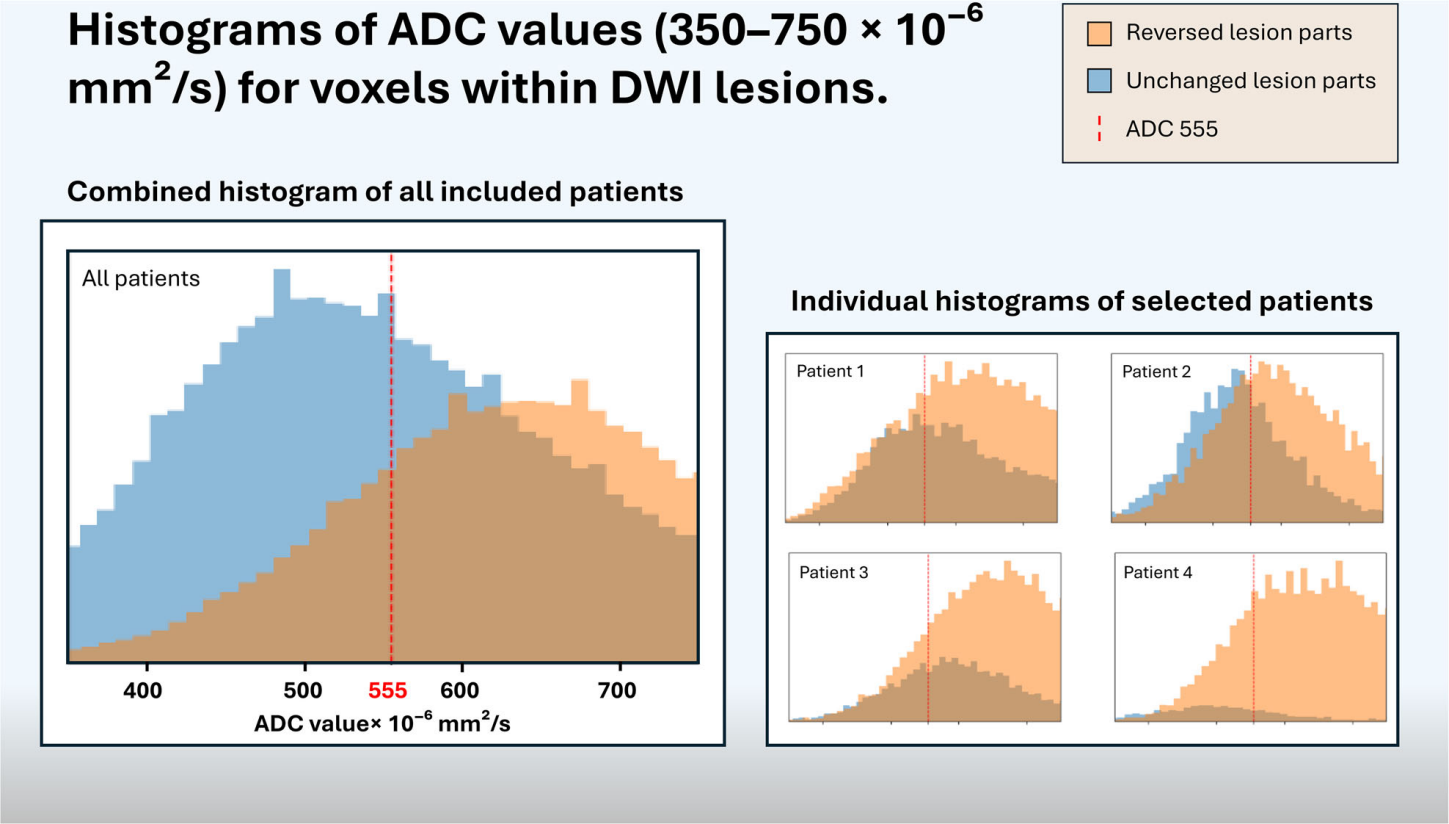
Histograms of DWI lesions. Each panel shows the distribution of ADC values in reversed (yellow) and unchanged (blue) voxels, ranging from 350 to 750 × 10^−6^ mm²/s, with higher values to the right. A vertical red line marks the “optimal” threshold of ADC = 555 × 10^−6^ mm²/s. Despite a general trend toward higher ADC in reversed tissue, the degree of overlap in most patients illustrates that an absolute cutoff often fails to segregate salvageable and irreversible tissue. The large panel compiles all included voxels from included patients into a single combined histogram, reinforcing the broad distributions for both reversed and unchanged lesions. The four smaller panels represent histograms from four individual patients. These overlapping histograms underscore the complexity of tissue fate in acute DWI lesions, suggesting that a single, universal ADC threshold cannot fully capture tissue viability

The ability of ADC to predict DWI-R was evaluated by ROC analysis, resulting in an area under the curve (AUC) of 0.708 (95% CI: 0.707–0.710) (Fig. 3). Using Youden’s J statistic, an ADC threshold of 555 × 10^–6^ mm^2^/s provided the optimal discrimination between voxels that reversed and not; at this threshold, sensitivity for detecting DWI-R was 73.8%, with a specificity of 58.6%. Sensitivity and specificity for various ADC thresholds are provided in Table 2. The use of ADC threshold as a prognostic test for DWI-R is illustrated in Fig. 4. The distribution of ADC values in reversed lesion parts is illustrated in Fig. 5, and histograms of ADC values in voxels associated with reversed and unchanged segments are provided in Fig. 6 and Supplementary Material.

An analysis of a subgroup including only anterior circulation occlusion with full recanalization (mTICI 3) within 90 min from MRI (20 patients), gave no improvement in discriminating power with AUC of 0.685 (95% CI: 0.683, 0.688), sensitivity 69.4%, specificity 58.9% and optimal threshold being 570 × 10^−6^ mm²/s.

## Discussion

In this study, we evaluated how well an ADC threshold could predict DWI-R in patients who achieved rapid and successful recanalization with EVT. Our main finding was that although voxel-level ADC values differ between reversed and non-reversed lesion segments, the discriminative power of a single ADC threshold before treatment is modest. When using Youden’s J statistic, an optimal threshold of 555 × 10^−6^ mm^2^/s led to a sensitivity of approximately 73.8% and a specificity of only about 58.6% (AUC = 0.708). Also, the predominantly overlapping histograms for reversed and unchanged DWI lesions (Fig. 6) highlight the limited ability of a single cutoff to distinguish salvageable from irreversibly damaged tissue. Although reversed voxels tended to cluster around slightly higher ADC values, the overlap was substantial. The minor separation in the peaks of reversed vs unchanged lesion parts, shows that voxels with identical ADC values often have different fates, while the broad shape of each distribution highlights considerable variability within each subgroup.

We also explored whether there is a lower ADC boundary below which DWI-R does not occur. While lower ADC values markedly reduced the probability of reversal, they did not eliminate it (Fig. 2 and Table 2). For example, at the cutoff of 620 × 10^−6^ mm²/s, which is frequently cited but not extensively validated [9], the probability of reversal for individual voxels was 49%, and 28% of all voxels below this threshold still reversed. Even at a threshold as low as 520 × 10^–6^ mm^2^/s [5, 7], the probability for reversal for individual voxels was 29% and 18% of all voxels below this level reversed. The chance of reversal became negligible at very low ADC levels (350 × 10^–6^ mm^2^/s and below). Although this might suggest a boundary, such instances represented a very small fraction of the total lesion volume. Therefore, our findings suggest that while lower ADC correlates with a reduced chance of reversal, a strict universal lower ADC boundary is not evident across the main range of values encountered in clinical practice.

DWI-R may not be solely determined by ADC but also influenced by clinical factors such as lesion site, time to recanalization, collateral status, and patient-specific physiological variability [26–29]. Our subgroup analysis, restricted to patients who achieved complete recanalization (mTICI 3) within 90 min, showed a slight decrease in discriminative power. This finding suggests that rapid and complete reperfusion may enhance tissue salvage even at low ADC values, whereas tissue with higher values often still fails to recover. The optimal ADC threshold identified in our analysis (555 × 10^−6^ mm²/s) is close to, yet lower than, the commonly referenced core threshold (620 × 10^−6^ mm²/s) established in patients receiving thrombolysis [9]. This further supports the notion that successful, aggressive treatment increases the likelihood of lesion reversal even at low ADC values.

A key limitation of using a single ADC threshold is variability in normal brain ADC values, grey matter generally has higher ADC than white matter [11]. This heterogeneity can transform into DWI lesions and undermine a uniform cutoff (Fig. 5). Consequently, a single cutoff applied uniformly across the brain may prove insufficient, and intra-brain heterogeneity should not be overlooked when interpreting ADC values in acute stroke, especially when considering high-stakes treatment decisions [26]. McArthur et al recently demonstrated that applying an ADC threshold of 620 × 10^−6^ mm²/s resulted in a few instances of ‘ghost infarcts’, defined as cases where the estimated infarct core exceeded the final infarct volume by more than 10 mL [30]. While volume-based assessments provide valuable information, our results underscore the added value of voxel-level analyses, which can more precisely capture the complex and heterogeneous tissue dynamics in acute ischaemic stroke, occurring both before and after recanalization. Specifically, simultaneous shrinking and expansion of different lesion areas may yield minimal net volume changes, or even volume increases, despite meaningful tissue salvage that would likely have contributed to a larger final infarct without EVT [31]. Our findings align with several previous studies highlighting the limitations of relying on a single ADC threshold to accurately predict tissue viability [32–36]. From a pathophysiological perspective, the reduction in ADC observed in acute ischaemic stroke is typically attributed to restricted water diffusion secondary to cytotoxic oedema [37]. This oedema arises from cellular energy failure, which disrupts ionic gradients and drives osmotic water influx into cells. A proposed early mechanism involves aquaporin-4-mediated astrocytic swelling, which rapidly reduces extracellular space and restricts water mobility, leading to low ADC values even before irreversible neuronal injury occurs [38].

A large body of evidence supports EVT in patients with large vessel occlusion early after stroke onset, regardless of infarct size [39, 40]. Our study reinforces this by underscoring the challenges of predicting tissue outcome using advanced imaging, including MRI, which is often considered the best available technique. This highlights the clinical imperative to prioritise timely treatment over additional imaging, especially when MRI may delay access to EVT. Although our findings suggest that a fixed ADC threshold alone has limited clinical value for accurately distinguishing salvageable from unsalvageable tissue, this does not negate the utility of DWI, particularly in challenging cases in extended time windows. Rather, it highlights the importance of interpreting ADC values in the context of patient-specific factors such as time since stroke onset, lesion size and location, and collateral status, as well as other clinical information and complementary imaging findings [41, 42]. To improve predictions, future studies should use normalised ADC measures and stratify data by tissue type to account for baseline differences.

The measured signal behaviour in diffusion-weighted MRI reflects a multi-compartment process where each image voxel represents the combined signal from thousands of brain cells and their surroundings [43]. The measured ADC values depend on tissue-specific properties, as well as sequence-specific factors, such as diffusion gradient strength and gradient timing schemes [44]. Even when identical sequence parameters are applied, ADC measurements may still vary slightly across MRI scanners from different vendors and models [45, 46]. These scanner-dependent differences may result from variations in both hardware components and post-processing methods. To reduce the impact of such non-biological variability, we included only patients imaged on identical MR systems using the same maximum *b*-value. Additionally, rigorous image co-registration and segmentation techniques were applied to our relatively large cohort with high-quality MR images, thereby reducing random and systematic errors. Nonetheless, technical challenges such as partial volume effects from co-registration, motion artifacts, and potential segmentation biases remain. The use of a 1 mL volume threshold and exclusion of voxels with ADC > 760 × 10^−6^ mm²/s helped mitigate these issues, but the limited number of voxels in the lower ADC range introduces some uncertainty, particularly regarding the lower limit for DWI-R. While our study reflects a real-world acute stroke setting, we specifically included patients with rapid and successful recanalization, meaning DWI-R may be less favourable in a more heterogeneous stroke population. Furthermore, the single-centre design and use of a single MR scanner may limit the generalizability of our findings, particularly regarding the precision of the optimal ADC threshold. Further limitations include the retrospective design and the fact that most of our patients had modest lesion sizes, with inclusion occurring before the benefit of EVT in large-core infarcts was established in randomised controlled trials. This could potentially limit the generalizability of our findings to more recent stroke populations. Additionally, the study relies on imaging endpoints without histological validation or direct correlation to functional outcomes. Lesion fate was defined by DWI signal 12–36 h after reperfusion, which may not fully capture tissue viability. Although prior studies have demonstrated a relationship between DWI signal and clinical outcomes [4, 29, 47], our findings should be validated in larger cohorts with clinical endpoints to determine the true clinical relevance and limitations of using a universal ADC threshold.

In conclusion, although reversed lesion parts exhibited marginally higher mean ADC values than persistent ones, a single ADC threshold yielded only moderate sensitivity and low specificity for predicting DWI-R in patients successfully treated with EVT. We did not identify a lower ADC boundary below which lesion reversal would become negligible across clinically relevant ranges. These findings underscore the complex nature of DWI-R and the limitations of a one-size-fits-all thresholding strategy.

## Supplementary information

ELECTRONIC SUPPLEMENTARY MATERIAL

## Abbreviations

ADC: Apparent diffusion coefficient
AUC: Area under the curve
DWI: Diffusion-weighted imaging
DWI-R: Diffusion-weighted imaging lesion reversal
EVT: Endovascular therapy
MRI: Magnetic resonance imaging
mRS: Modified Rankin scale
mTICI: Modified treatment in cerebral infarction score
ROC: Receiver operating characteristic
