# Supplementary material for: Predicting lesion reversal in acute cerebral ischaemia via apparent diffusion coefficient threshold on diffusion-weighted MRI
Source: Eur Radiol. 2025 Sep 29;36(3):1733–43. doi: 10.1007/s00330-025-12014-0 (PMC12963182; doi:10.1007/s00330-025-12014-0)
Supplement: Supplementary file 1 — ELECTRONIC SUPPLEMENTARY MATERIAL [file 330_2025_12014_MOESM1_ESM.pdf]

Predicting Lesion Reversal in Acute Cerebral Ischemia via Apparent Diffusion Coefficient Threshold on Diffusion-weighted MRI

ELECTRONIC SUPPLEMENTARY MATERIAL

Eur Radiol (2025) Skattør TH, Bjørnerud A, Nome T, et al.

Figure S1. Histograms of diffusion-weighted imaging (DWI) lesions from all included patients. Each panel shows the distribution of apparent diffusion coefficient (ADC) values in reversed (yellow) and unchanged (blue) voxels ranging from 350 – 750 × 10^{-6} mm²/s with higher values to the right. A vertical red line marks the "optimal" threshold of ADC = 555 × 10^{-6} mm²/s. Despite a general trend toward higher ADC in reversed tissue, the degree of overlap in most patients illustrates that an absolute cutoff often fails to segregate salvageable and irreversible tissue. The first panel compiles all included voxels from all patients into a single combined histogram.

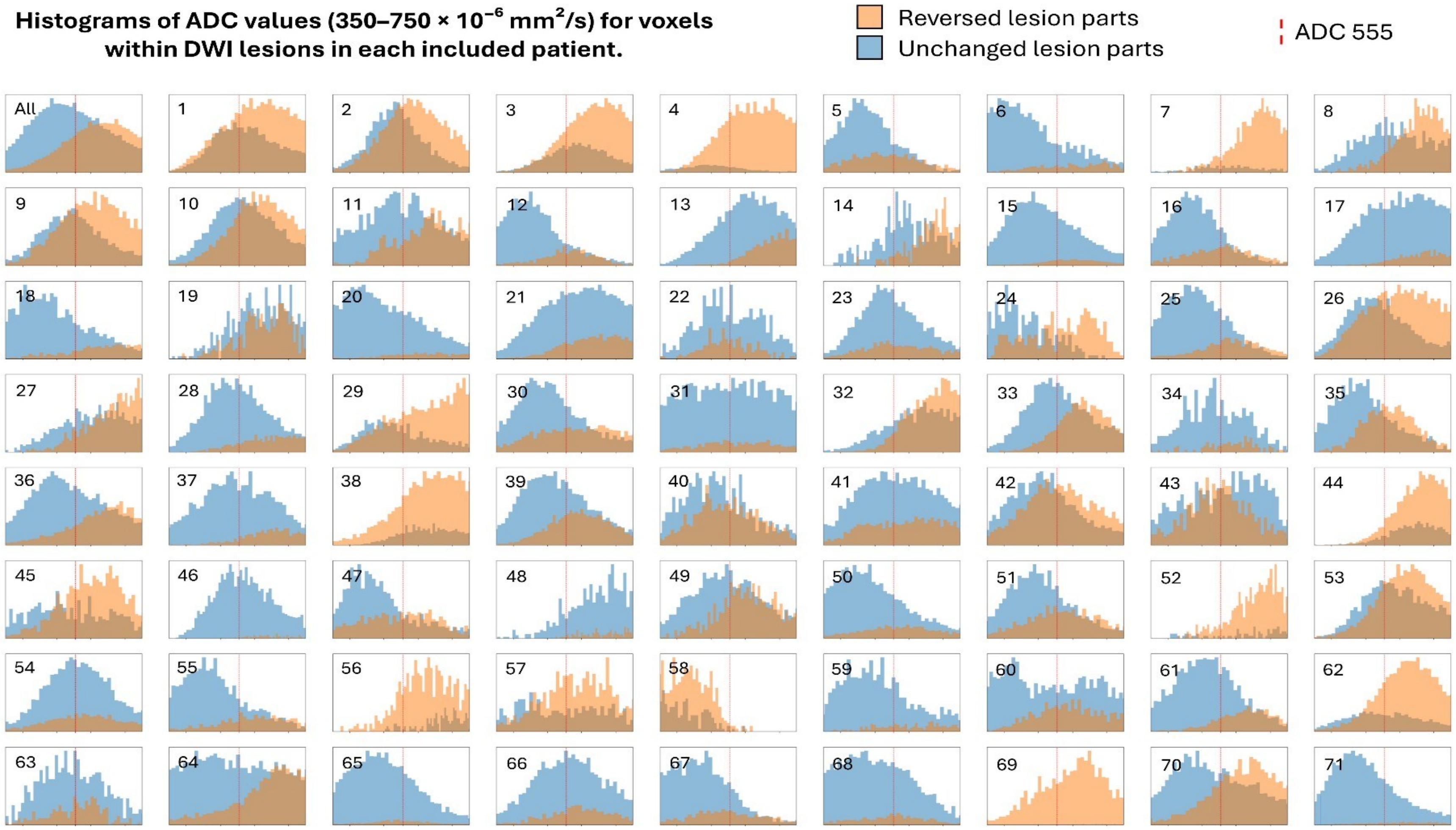

Table S1. Overview of MRI sequences performed pre- and post-EVT.
The first column lists all sequences included in the standard imaging protocols. Pre-EVT: Diffusion-weighted imaging (DWI), fluid-attenuated inversion recovery (FLAIR), time-of-flight angiography (TOF), susceptibility-weighted imaging (SWI), and dynamic susceptibility contrast perfusion (DSC). Post-EVT: DWI, T2 turbo spin echo (T2 TSE), FLAIR, TOF, SWI, and T1-weighted magnetization-prepared rapid gradient echo (T1).

The second column indicates the number of patients who underwent each sequence, with the percentage of the total cohort in parentheses. Pre-EVT sequence selection varied based on clinical indication and the judgment of the treating interventional neuroradiologist. Post-EVT variation was primarily due to the patient's clinical condition (e.g., inability to lie still).

Variation in DWI sequence parameters is described in the main text. Minor variations in parameters of other sequences are not reported, as they were not considered relevant to the analyses conducted in this study.

Table S1. Overview of MRI sequences performed pre- and post-EVT

MRI Sequence	Patients, n (%)	Echo Time, ms	Repetition Time, ms	Flip Angle, °	Matrix	Field of View, mm	Slice Thickness, mm
**Pre-EVT**							
DWI	71 (100)	90	6400	90	384×384	229×229	6.5
FLAIR	57 (80.3)	98	9700	150	256×256	230×230	5
TOF	55 (77,5)	7	24	20	512×496	174×180	0.7
SWI	52 (73.2)	30	39	15	256×232	208×230	3
DSC	29 (40.8)	30	1610	90	128×128	230×230	5
**Post-EVT**							
DWI	71 (100)	90	6400	90	384×384	229×229	6.5
FLAIR	69 (97.2)	337	5000	120	512×480	240×256	1
TOF	69 (97.2)	7	24	20	512×496	174×180	0.5
SWI	69 (97.2)	40	49	15	512×464	208×230	2
T2 TSE	64 (90.1)	86	4780	139	896×812	208×230	4
T1	60 (84.5)	2.4	1900	10	460×460	256×256	1
